# Unveiling the role of higher education institutions in regional sustainability transitions: a systematic literature review and research agenda

**DOI:** 10.1007/s11625-025-01736-1

**Published:** 2025-09-04

**Authors:** Ines Hinterleitner, Gesa Pflitsch, Marianne Penker, Sabine Sedlacek, Verena Radinger-Peer

**Affiliations:** 1 Institute of Landscape Development, Recreation and Conservation Planning, BOKU University, Peter-Jordan-Straße 65, 1180 Vienna, Austria; 2 Institute of Sustainable Economic Development, BOKU University, Feistmantelstraße 4, 1180 Vienna, Austria; 3 School of Sustainability, Governance and Methods, Modul University Vienna, Am Kahlenberg 1, 1190 Vienna, Austria

**Keywords:** University roles, Regional sustainability transitions, Role theory, Systematic literature review, University-community engagement

## Abstract

Due to pressing sustainability challenges, our society is in urgent need for innovations and new knowledge. Higher education institutions (HEIs) as institutions of education and knowledge production are attributed a leading role in the sustainability endeavor. In recent years, the topic of the contributions of HEIs to regional sustainability transitions (RST) has received increasing attention, resulting in rising numbers of literature published. However, due to its position at the interface of different research fields, the knowledge on the role of HEIs in RST is heterogeneous and scattered. This paper provides a systematic review of the literature on HEIs’ role in RST from 2007 onwards. The purpose of the paper is to identify how this role is conceptualized and empirically investigated and to understand better which challenges HEIs are facing and what kinds of drivers enable them to take over this role. The results are aligned along with role characteristics (actors, activities, drivers, challenges, and impacts), which provide in-depth insights into how this role is conceptualized. The authors deduced one role of HEIs in RST with three dimensions from the existing literature: Sustainable Regional Operations, Community Empowerment, and Regional Sustainability Policy. HEI students, faculty, leadership and management as well as a diverse range of regional actors are engaged in the enactment of this role and its three dimensions through educational, research, and outreach activities. However, these activities are rarely institutionalized and therefore depend on the engagement of individuals and the support of the HEI leadership. Based on these results, the authors propose further avenues of research which have the potential to better characterize the role of HEIs in RST including the perspective of who decides within HEIs on the role and the evolution of this role over time.

## Introduction

As sustainability challenges have become more pressing in recent years (Richardson et al. 2023), higher education institutions^1^ (HEIs) are increasingly oriented toward sustainability. Scholars worldwide have noted that the advancement of sustainable development (SD) in HEIs has shifted from a ‘Sustainability in Higher Education’ toward a ‘Higher Education for SD’ phase. In the former, HEIs integrated SD into curricula (Cortese 2003; Lozano et al. 2013), research (Waas et al. 2010), management practices (Lozano 2006), and campus operations (Leal Filho 2011), while the latter is characterized by knowledge co-production with external stakeholders and the aim to contribute to more fundamental transitions to sustainability (Dlouhá et al. 2013; Trencher et al. 2014a; Zilahy and Huisingh 2009). Additionally, in this latter phase, HEIs started to focus their engagement increasingly on their surrounding society, particularly their local communities and regions (Trencher et al. 2014a).

In recent years, this emerging phenomenon of HEIs engaging in sustainability transitions within their regional contexts has gained increasing attention, visible in rising publication numbers since 2007. Thus far, this emerging phenomenon has mainly been explored through three distinct lenses: HEIs, sustainability, and region. Each lens highlights different aspects at the intersections between the phenomenon’s core dimensions. Due to the different lenses and the associated diversity of aspects used in studying HEIs’ contributions to regional sustainability transitions (RST), there is a wide variety of roles attributed to HEIs in RST: They are regarded as change agents, intermediaries, connectors, coordinators, mediators, co-producers, facilitators, investigators, catalysts, anchor institutions, or boundary spanners for RST (Devine-Wright et al. 2001; Mdleleni 2022; Peer and Stoeglehner 2013; Pflitsch and Radinger-Peer 2018). A more systematic conceptualization of this increasing amount of research is needed to better understand the role(s) of HEIs in RST and to contextualize HEIs in their specific regional context more systematically.

Based on a systematic literature review and employing a role theoretical framework, the present paper therefore aims to address the following research question:

How does the role/do the roles of HEIs in RST manifest themselves theoretically and empirically in the existing literature?

This research question is specified by the following sub-questions:

- How is this role/are these roles conceptualized and what theoretical approaches are utilized?
- How is this role/are these roles described empirically in terms of actors and activities?
- What impacts of HEIs on RST are depicted in literature?
- Which drivers and challenges have been found regarding this role/these roles?
- How did the role(s) of HEIs in their regional context change over time?

The paper is structured as follows: The section “The role(s) of HEIs in RST: an analytical framework” introduces literature on roles of HEIs and role theory as the theoretical framework employed in this paper. The section “Methodology” describes the methodological approach of the systematic literature review and content analysis. Thereafter, the section “Results” presents the results of the systematic review. The section “Discussion and further avenues of research” discusses the results and identifies further avenues for research. The final section presents the section “Conclusion”.

## The role(s) of HEIs in RST: an analytical framework

This chapter introduces literature on roles of HEIs which is followed by role theory as the theoretical framework guiding the analysis and interpretation of the systematic literature review.

### HEIs’ models and roles

HEIs are among the oldest organizations in Western society and are therefore regarded as institutions themselves (Diogo et al. 2015). Starting out as elitist teaching institutions in the eleventh century, they evolved into institutions where education and research are combined and influence each other. Up until the Second World War, disciplines were established as places of efficient knowledge production. During the Second World War, the first interdisciplinary research projects emerged, and scientists were solely regarded as contributing knowledge to difficult societal problems. Following the student protests in 1968, HEIs evolved from being rather elitist institutions to places for mass education and important cornerstones of a democratic society (Scholz 2020). This evolution shows that HEIs were regarded as knowledge creators, knowledge providers, and educators.

From the 1980s and 1990s on, with progressive functional differentiation of our society, we see the emergence of different HEI models. They try to capture and explain how HEIs contribute to society through their education and research activities and the newly established third mission (Radinger-Peer 2019).

HEI models focusing on regional development (i.e., Entrepreneurial University, Triple-Helix University, and Regional Innovation System University) attribute knowledge production, provision, and transfer as the main task to HEIs (Gunasekara 2006; Radinger-Peer 2019; Trippl et al. 2015; Uyarra 2010). Next to this focus on regional development, there are also HEI models which focus on societal development (i.e., Mode 2 University and Engaged/Civic University). These models attribute a more active role to HEIs in contributing to societal development and transformation through co-producing knowledge (Nowotny et al. 2003) and integrating regional needs into HEIs’ functions and missions (Goddard et al. 2016; Uyarra 2010). More recently, the Transformative or Sustainable University model has been proposed as a way to contribute to societal sustainability transformation (Loorbach and Wittmayer 2023; Schneidewind 2014; Schneidewind and Singer-Brodowski 2014; Trencher et al. 2014b) through integrating sustainability aspects into HEIs’ functions and activities (Arbo and Benneworth 2007; Loorbach and Wittmayer 2023; Schneidewind and Singer-Brodowski 2014) and engaging with societal actors on sustainability challenges (Trencher et al. 2014b). HEIs aim to become co-creators (Trencher et al. 2014b) or change agents for sustainability (Stephens et al. 2008).

It becomes apparent that HEIs are attributed a diversity of facilitating and mediating functions in regional, societal, or SD on the basis of their potential to link different sectors of expertise and networks (Arbo and Benneworth 2007; Sedlacek 2013), and to create and co-produce knowledge (Scholz 2020). To deepen the understanding of the role(s) of HEIs, role theory is now introduced as the analytical framework guiding this literature review.

### Role theory

Elaborations on role theory date back to the 1930s (Linton 1936; Mead 1934). It has been developed to guide the identification of ideal types of behavior and attitudes of individuals in certain social settings through the description of their characteristics (Biddle 1986). The main hypotheses of role theory are: (1) roles are comprised of a set of actors’ attitudes and behaviors (Linton 1936; Wittmayer et al. 2017), which are specific to a certain context (Biddle 1986; Mead 1934). Therefore, (2) roles can be viewed as socially constructed (Turner 1990; Wittmayer et al. 2017) and (3) they evolve as societal demands and context change (Wittmayer et al. 2017). Moreover, the concept of roles helps to establish “a shared reality to which actors can refer” (Wittmayer et al. 2017) and therefore provide a guide for a complex society.

Referring to Callero (1994), Hilbert (1981), Lynch (2007), and Turner (1990) who have contributed to the field of role theory, the authors derive the following characteristics from role theory for the analysis of the retrieved material:

- Roles are understood as social roles which comprise certain *regular patterns of behavior and attitudes* which are regarded as strategies for dealing with recurring sets of situations. Moreover, they are attributed certain rights and duties (Turner 1990).
- Roles are defined *in relation to other roles* (Lynch 2007), meaning that roles can change when the *environment around them changes*. Role change can be the creation of a new role or dissolving of an established role. This change in roles is induced through (1) change in cultural values; (2) demographic or technological change; (3) social structural change; or (4) due to dysfunctionality (Turner 1990). Roles can change quantitatively (e.g., through a quantitative change in rights or duties) or qualitatively (e.g., through a reinterpretation or its meaning or a change in one of its core elements) (Turner 1990).
- Roles are considered real features of the social world “as they are recognized, accepted, and used to *accomplish pragmatic interactive goals* in a community” (Callero 1994, p. 232).
- Roles are used to *gain access to cultural, social or material resources* (Callero 1994; Hilbert 1981) through which they become a *tool for agency and power* for social action (Callero 1994).
- Roles contribute to the *creation of social structures* while social structures influence the construction of social roles (Callero 1994).

### Application of role theory to HEIs

HEIs are large, multi-dimensional and complex and have decentralized structures, resulting in high autonomy of its individual members (Nieth and Radinger-Peer 2023). Due to this limited steering potential from the top, it is in particular the agency of individual HEI members which is key, despite the strategic and structural anchoring of the HEIs (Purcell et al. 2019). Both the institution and its individuals can be regarded as actors who have collective or individual agency (Avelino and Wittmayer 2016; Wieczorek and Hekkert 2012). In this paper, the authors aim to investigate the role(s) HEIs as an institutional actor play in RST. However, as it is often the individual members’ behavior and perspectives who provide entry points to understanding institutions (Gonzales et al. 2018), individual actors of HEIs are also taken into account in the following analysis.

### Operationalization of role theory for application in the context of HEIs in RST

As is written, e.g., in the Austrian University Act 2002 §1/1, Independent Expert Group on the Universities and the 2030 Agenda (2022) or UNESCO (2022), HEIs provide important contributions to solving humanity’s problems and the prosperous development of society and the natural environment (→* accomplish goals in a community/impact*). They achieve this through teaching, research, and outreach activities through which they educate, create, and diffuse knowledge (→ *regular patterns of behavior and attitude/activities*). These activities and attitudes of HEIs are context-specific, meaning that they change when societal structures change as has been demonstrated by Scholz (2020) who investigated HEIs’ roles and functions since their foundation. Moreover, as has been stated by neo-institutional (Manning 2017) and organizational (Gonzales et al. 2018) theory, HEIs as institutions are understood as embedded in a political and social environment which shapes their behavior and attitudes (→ *regular patterns of behavior and attitudes, influenced by changes in the environment, interrelationship with social structures/challenges and drivers*). On the basis of their social mandate (Scholz 2020) and the level of trust they enjoy (Trencher et al. 2014a), HEIs and its individual members have access to certain resources (DiMaggio and Powell 1991) which afford them both with autonomy and the capacity to exert influence over social structures (→* accomplish goals in a community, tools for power and agency/challenges and drivers, impact*).

**Fig. 1:**
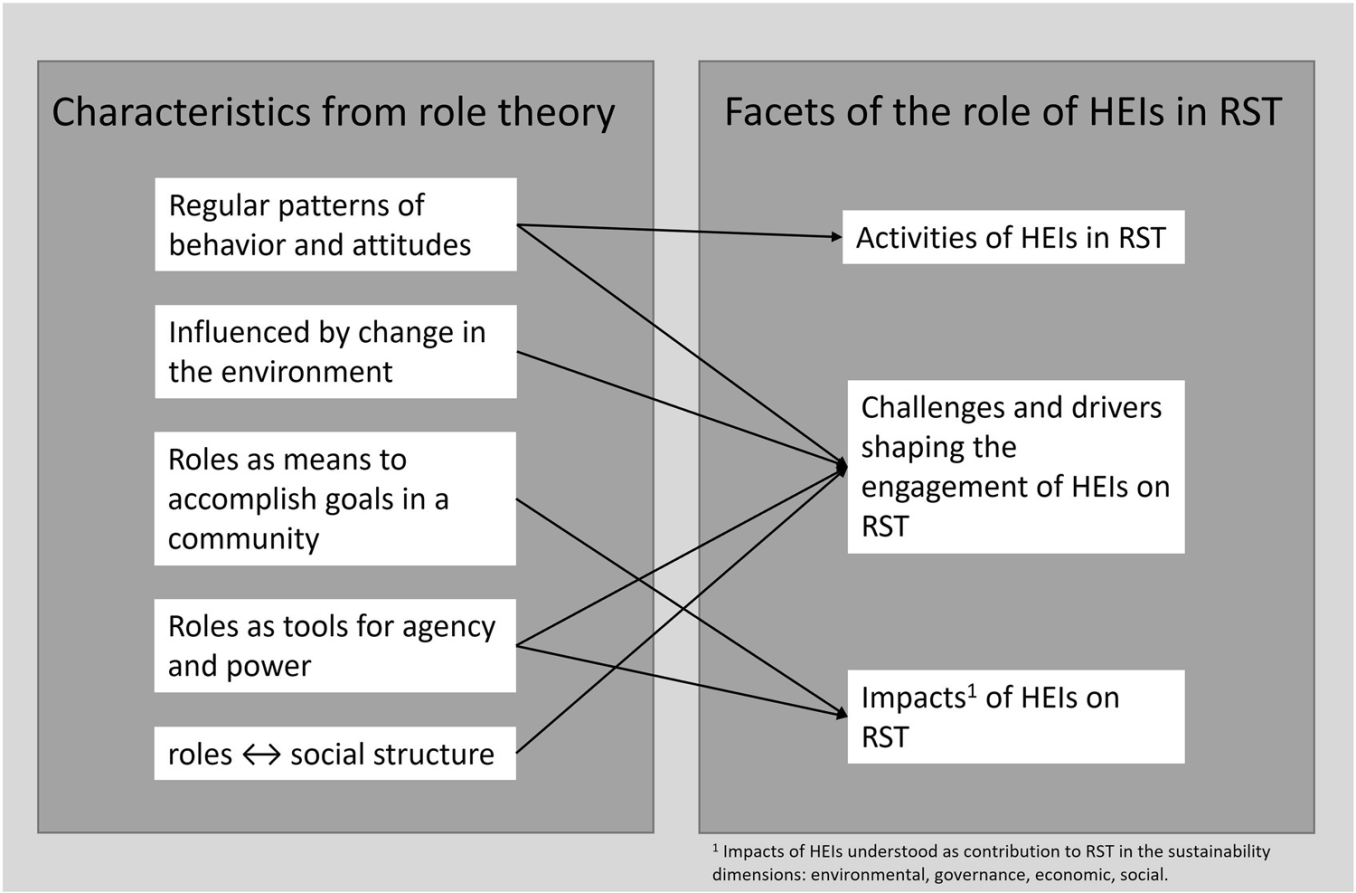
Depiction of main role characteristics from role theory and how they guide the analysis in this literature review [own illustration according to Callero (1994), Hilbert (1981), Lynch (2007) and Turner (1990)]

This is summarized in Fig. 1 which depicts the main role characteristics from role theory [based on Callero (1994), Hilbert (1981), Lynch (2007) and Turner (1990)] and how it is applied to the topic of HEIs in RST to guide the analysis in this systematic literature review.

## Methodology

### Literature identification and selection

The present study employs a systematic literature review protocol to identify relevant literature and conduct a quantitative and qualitative analysis. A systematic literature review is a valuable approach for defining the current state of knowledge on a research topic and identifying both advancements and gaps in the emerging literature (Denyer and Tranfield 2009).

To identify the relevant literature, the authors employed the search engines Scopus and Web of Science (WoS). After an iterative process of experimentation with synonyms and search operators around the terms “Higher Education Institutions”, “sustainability”, “region”, and “interaction”, the authors selected the following search string to balance precision and comprehensiveness: (“higher education institution” OR universit* OR college*) AND (“sustainab* transition*” OR “sustainab* transform*” OR “sustainab* develop*” OR “Agenda 2030”) AND (region* OR county OR city OR urban* OR place* OR local*) AND (interact* OR engage* OR co-creat* OR initiat* OR co-produc* OR co-design*). The search string was limited to the fields of Title, Abstract, and Keywords to ensure a focus on the specified selection criterium. The objective was to identify papers that specifically address the role of HEIs in regional SD and/or transitions. The first literature search was finalized in December 2023.

This review explicitly focuses on *regional and local (including semi-urban) scales* and therefore only includes papers to which this criterium applies. Additionally, only papers published in *peer-review journals* and in English language as the most used language in academic writing (Genç and Bada 2010) were included.

**Fig. 2:**
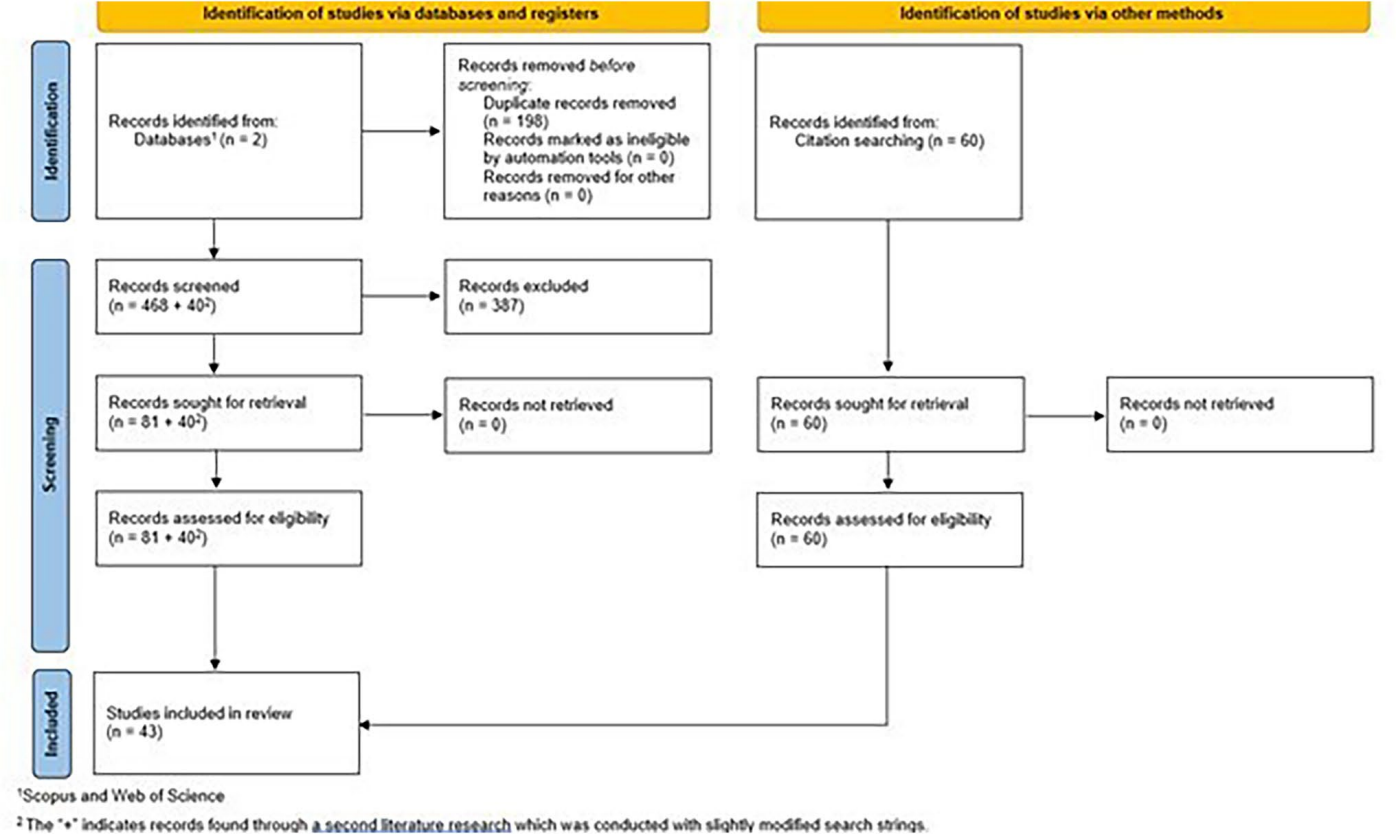
PRISMA flow diagram depicting the literature search process [own illustration based on Page et al. (2021)]

The selection of the sample follows the PRISMA guidelines for a systematic literature review (Page et al. 2021) (see Fig. 2). Following the removal of duplicates, a total of 486 texts were subjected to the assessment process for eligibility of inclusion in this review, comparing the research questions or objectives of the retrieved texts with the established selection criterion. After this initial screening, the full text of 81 papers was assessed for eligibility based on the established selection criterion.

Concurrently, a second literature search was conducted in February 2024 using again the databases Scopus and WoS. Based on the insights of the literature retrieved in the first round, two revised search strings were employed: (1) a fifth search term focusing on the term “societal challenges” and synonyms (i.e., “grand challenge*” OR “societal challenge*” OR “societal problem*” OR “cultural development” OR “social development” OR “ecological development” OR “environmental development” OR “societal innovation*” OR “social innovation*” OR “climate change challenge*”) was added and (2) the sustainability term was limited on regional and urban SD, (i.e., “sustainable regional development” OR “sustainable urban development”). Following the removal of duplicates, the authors assessed based on the selection criterion 29 texts of the first revised search string and 11 texts of the second revised search string for eligibility for inclusion in this study. Additionally, the authors undertook citation searching based on the retrieved literature which yielded 60 texts. These 60 texts were again subjected to an assessment for eligibility based on the established selection criterion.

The full-text assessment of the retrieved texts according to our selection criterion led to 43 papers being subsequently subjected to quantitative and qualitative analysis (see Appendix A for an overview of the analyzed literature). To ensure the reliability of the systematic sampling procedure process and validity of results of the subsequent content analysis, all steps were carried out in close interaction of the first and corresponding author.

See Fig. 2 for a graphical overview of the process of literature search according to the PRISMA approach (Page et al. 2021).

### Analytical steps for quantitative and qualitative analysis

For the descriptive analysis of the retrieved texts, the authors utilized the following codes: “journal”, “year”, “method”, “scale”, and “place”.

The qualitative analysis of the sample is based on the operationalization proposed by Wittmayer et al. (2017) (see also section “Operationalization of role theory for application in the context of HEIs in RST”, Fig. 1). Accordingly, the authors pre-defined three main coding categories and associated sub-codes based on the research questions and the analytical framework to identify different aspects of the role(s) of HEIs in RST. Following the initial coding of all papers, the coding categories were revised, resulting in the coding scheme presented in Table 1. The final coding scheme was employed to re-code all texts, using the qualitative data analysis software Atlas.ti (ATLAS.ti Scientific Software Development GmbH 2024).

**Table 1:** Overview of main and sub-coding categories for the content analysis of the material (own illustration)

Conceptualization of role	Empirical expression of role	Regional influencing factors
Theoretical approaches	Activities	Challenges
Conceptualization of role	Involved actors	Drivers
Definition of role	Sustainability impacts	

The aim of the coding was to identify all relevant aspects for identifying the role(s) of HEIs in RST. Consequently, following the completion of the coding process, the main code “conceptualization of the role” was clustered around emerging topics and its three sub-codes are presented together in the section “Results”. The sub-codes “activities”, “involved actors” and “sustainability impacts” were also clustered around emerging topics within these sub-codes and are presented separately in the section “Results”. The code “regional influencing factors” is depicted in the section “Results” in accordance with the sub-codes “challenges” and “drivers”.

With the expectation to derive different roles or dimensions of a role, the identified activities, actors, challenges, drivers, and impacts were clustered according to the conceptual models used in the text (e.g., entrepreneurial university and engaged university) in a first step. However, these clusters showed large overlaps in all areas (i.e., actors, activities, challenges, drivers, and impacts). The author therefore decided to investigate the coded material for the distinguishing aspects: it turned out that the code “impact” revealed the highest divergence. Therefore, the clusters describing different roles or dimensions of one role (see the section “Discussion and further avenues of research”) were built around the impacts of HEIs to RST.

## Results

This section starts with a short overview of the descriptive results of the sample and then proceeds to depict the results of the qualitative content analysis of the material.

### Descriptive overview of the sample literature

**Fig. 3:**
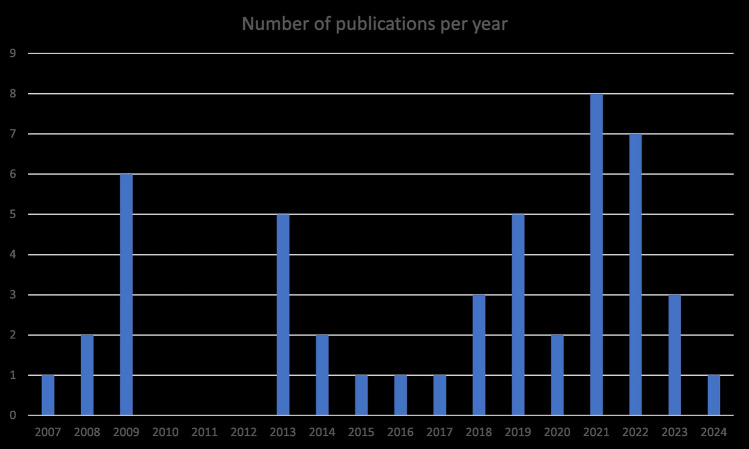
Year of publication (own illustration)

Figure 3 shows the years of publication of the sample. It dates back to 2007, demonstrating the rather recent interest in this research field.

Regarding the geographical scale, most of the papers included apply a regional perspective, comprising several communities, towns, cities, or districts of a nation. There are a few who take on a semi-urban or urban (Anderson et al. 2013; Bogedain and Hamm 2020; Málovics et al. 2022; Martínez-Acosta et al. 2023; Neary and Osborne 2018; Quest et al. 2019; Rosenberg Daneri et al. 2015; Shiel et al. 2016) perspective, which Bogedain and Hamm (2020) also consider local. Two papers mention the local level (neighborhood or municipality) as their geographical scale (Leal Filho et al. 2019; Paunović et al. 2022).

The most frequently used outlets included in the presented systematic literature review are the Journal of Cleaner Production (11 texts), Sustainability (8 texts), and the International Journal of Sustainability in Higher Education (6 texts). The remaining 23 texts of the sample were published in journals which occur only once (for an overview, refer to Appendix B, Table 3).

### The role(s) of HEIs in RST: results of the qualitative analysis

#### Conceptualization of the role(s) of HEIs in RST

Building on the literature on the role of HEIs in regional development in general and the knowledge society in particular [see Compagnucci and Spigarelli (2020), Trippl et al. (2015), Uyarra (2010)], many publications in the sample use the concept of *Third Mission* (or even Fourth Mission) to describe the societal role or mission of HEIs that goes beyond their traditional tasks in teaching and research (Adamakou et al. 2021; Agusdinata 2022; Bogedain and Hamm 2020; Budowle et al. 2021; Hoinle et al. 2021; Lehmann et al. 2009; Mancini et al. 2022; Marchigiani and Garofolo 2023; Martínez-Acosta et al. 2023; Mbah 2019; Mdleleni 2022; Neary and Osborne 2018; Paunović et al. 2022; Peer and Stoeglehner 2013; Pflitsch and Radinger-Peer 2018; Velasco et al. 2021; Wakkee et al. 2019). Along with this, references are made to the different *university models* that have been proposed in this literature, which each offer distinct conceptualizations of HEIs’ societal responsibilities and activities.

The *Engaged University* model is employed in several papers [in particular, references are made to the seminal papers by Chatterton and Goddard (2000) and Breznitz and Feldman (2012)] as a starting point to conceptualize HEI’s role in RST (Adamakou et al. 2021; Hoinle et al. 2021; Leal Filho et al. 2019; Mancini et al. 2022; Mdleleni 2022; Pflitsch and Radinger-Peer 2018; Rosenberg Daneri et al. 2015; Sedlacek 2013; Velasco et al. 2021; Zilahy and Huisingh 2009). Following this work, the latter is described as that of a *facilitator or amplifier* [(Adamakou et al. 2021)**;** “catalyst” (Mdleleni 2022), “promoter and amplifier” (Velasco et al. 2021)] of social (innovation) processes. The choice of this model for examining the role of HEIs in the context of RST is justified by the fact that it is more strongly oriented toward social, explicitly regional needs than previous models and deems the cooperation with a broader range of regional actors (also including civil society) necessary (e.g., Adamakou et al. 2021; Pflitsch and Radinger-Peer 2018).

On the contrary, the *Entrepreneurial University* model is often used as a reference point to contrast how the role of HEIs was previously conceptualized and to highlight its limitations in the context of RST (Bogedain and Hamm 2020; Hoinle et al. 2021; Málovics et al. 2022; Mbah 2019; Pflitsch and Radinger-Peer 2018; Trencher et al. 2013, 2014a, b; Wakkee et al. 2019). Yet, a few papers explicitly use this model to emphasize that HEIs can play a key—*quite proactive*—role in shaping sustainable futures by fostering innovation and entrepreneurship in their regional environment (Martínez-Acosta et al. 2023; Paunović et al. 2022; Sedlacek 2013). However, in these cases, it is also pointed out that a broadening of the classic model of the Entrepreneurial University is necessary, in the sense that entrepreneurship needs to be understood in this context as a generic attitude or mindset that is not necessarily profit-oriented (Wakkee et al. 2019).

The *Transformative University* model which emphasizes that the “co-creation” of solutions with external stakeholders (should) take(s) on the status of a new fourth mission of the HEI (Trencher et al. 2013, 2014a, b) is adopted as a conceptual framework in several publications in the sample (Agusdinata 2022; Bogedain and Hamm 2020; Croese and Duminy 2023; Neary and Osborne 2018; Purcell et al. 2019; Rosenberg Daneri et al. 2015; Trencher et al. 2013, 2014a, b; Wakkee et al. 2019). In addition to moving away from the commercialization focus of earlier models, this model not only deems the co-production of knowledge between stakeholders necessary, but also the co-creation, i.e., joint implementation, of solutions. Based on this framework, the role of HEIs in RST is thus conceptualized as quite proactive, which is expressed in terms, such as “engine” (Agusdinata 2022; Purcell et al. 2019), “change agent” (Purcell et al. 2019), and “social transformer and co-creator” (Neary and Osborne 2018; Trencher et al. 2014b).

In addition, there are several publications in the sample that discuss the role of HEIs in RST—rather detached from the above literature—under the broader umbrella term of *Community Engagement* (Aluko and Okuwa 2018; Leal Filho et al. 2019; Málovics et al. 2022; Mbah 2019; Quest et al. 2019; Shabalala and Ngcwangu 2021; Shiel et al. 2016). In these publications, the concepts of Capacity Building [building on work by Brown et al. (2001), Groot and van der Molen (2000) and Spoth et al. (2004)] and Empowerment [by Kabeer (1999)] are frequently mobilized to analyze (1) how HEIs help to strengthen the capacities of regional actors and communities to achieve sustainability goals themselves or (2) how HEIs are strengthening individuals’ ability to make strategic life decisions. In addition to the moderating and mediating role, that HEIs are also ascribed in the Engaged University literature, the focus here is specifically on HEIs’ contribution to RST in terms of empowering society or individuals to develop independently.

#### Involved HEI and regional actors in RST

Besides HEI actors, there is a range of regional actors (public, economic, civil society, foundations) involved in engaging HEIs with RST. They are further elaborated below. For a comprehensive overview of the actors involved with the associated literature see Table 4 in Appendix B.

*HEI students* support data gathering in communities for research purposes (Bogedain and Hamm 2020; Trencher et al. 2014a), act as knowledge brokers in local communities (Bodorkós and Pataki 2009; Bogedain and Hamm 2020), or create solutions to local problems within their studies (Agusdinata 2022). Moreover, *student organizations* engage with sustainability transformations outside of HEIs through the initiation of projects with stakeholders in the region (Chang 2022) or initiating HEI-regional collaborations (Lukman et al. 2009; Wakkee et al. 2019). Perales Franco and McCowan (2021) highlight HEIs **alumni** as actors contributing to the RST of the community, e.g., through projects they started as students.

*Faculty, singular research groups/institutes* and *HEIs’ teachers* are mentioned as *initiators* of the engagement of HEIs with regional actors and RST, e.g., through asking for community land for teaching and research purposes (Aluko and Okuwa 2018), conducting research in the area (Bodorkós and Pataki 2009), initiating public engagement projects (Marchigiani and Garofolo 2023), or data gathering on a local challenge (Quest et al. 2019).

*HEIs’ management* and *leaders* (e.g., rectorate, dean) also influence HEIs’ engagement with regional actors through enabling the establishment of organizational and institutional structures for the engagement at the HEIs (Mader et al. 2008; Velasco et al. 2021). *HEIs’ administration* supports in legal questions (Paunović et al. 2022) and coordination of actors and activities (Trencher et al. 2014b). *HEIs’ service centers* such as knowledge exchange or transfer offices take on the task of coordinating regional sustainability networks (Lehmann et al. 2009) and sustainability committees/councils help creating climate action plans for the community (Budowle et al. 2021). *Regional Centers of Expertise* (RCEs) as a bridging entity between society and HEIs can be based at HEIs (Mader et al. 2008; Sedlacek 2013) where they initiate HEIs–region collaborations and contribute to awareness raising for SD among society, and HEIs’ staff and students (Mader et al. 2008).

Regional actors have been clustered into public, economic, and civil society to account for the diverse range of actors mentioned. They are presented exemplary: *Public actors* (i.e., governments, public administration, and institutions such as schools or churches) initiate HEIs’ engagement with RST through providing funding (Anderson et al. 2013; Marchigiani and Garofolo 2023), organizing workshops where regional challenges are being discussed (Bodorkós and Pataki 2009), or providing a link between HEIs and local/regional actors (Bogedain and Hamm 2020). *Economic actors* such as companies contribute to HEIs’ engagement with RST through providing financial resources and materials for research and education (Agusdinata 2022; Lukman et al. 2009). Moreover, private companies are the recipients of HEIs’ output such as new technology or knowledge on green production processes (Lukman et al. 2009). *Civil society actors* are comprised of individual citizens, community groups, associations and NGOs. Local communities contribute knowledge to research on local challenges, they are also the recipients of solutions proposed by HEI actors on local challenges (Agusdinata 2022). NGOs provide human resources for helping HEIs achieve their goals in a local community (Aluko and Okuwa 2018) and are also the result of the engagement of HEIs with regional challenges (Bodorkós and Pataki 2009). Finally, *foundations* have been mentioned and are listed separately here as they are not further specified and therefore difficult to attribute to one of the three categories. Foundations are important sources for funding projects related to RST (Aluko and Okuwa 2018; Bodorkós and Pataki 2009; Mancini et al. 2022; Velasco et al. 2021).

#### Activities of HEIs in RST

The activities of HEIs in RST are presented according to the three missions of HEIs: education, research, and outreach. Regarding education, the authors found that different teaching approaches (e.g., transdisciplinary, project-based or problem-oriented education) are utilized to foster regional engagement toward sustainability (Budowle et al. 2021; Hoinle et al. 2021; Lehmann et al. 2009; Perales Franco and McCowan 2021; Ramos 2009; Rosenberg Daneri et al. 2015; Velasco et al. 2021). Moreover, student internships (Budowle et al. 2021; Leal Filho et al. 2022; Mancini et al. 2022; Quest et al. 2019; Rosenberg Daneri et al. 2015; Shiel et al. 2016), degree theses (Budowle et al. 2021; Lukman et al. 2009; Shiel et al. 2016), and self-organized student projects (Chang 2022) are a way through which students engage with regional actors and contribute to RST. Next to different teaching approaches, different research approaches (e.g., transdisciplinary research, citizen science, participatory action research, or collaborative research) are mentioned through which researchers engage with regional actors and regional challenges (Bogedain and Hamm 2020; Málovics et al. 2022; Quest et al. 2019; Shiel et al. 2016; Trencher et al. 2013). Finally, outreach activities are a way to engage with regional actors. These activities exhibit a diversity, ranging from facilitating exchange between regional actors (Bodorkós and Pataki 2009; Croese and Duminy 2023; Mader et al. 2008), participating in regional sustainability networks (Lehmann et al. 2009; Quest et al. 2019), providing policy briefs (Neary and Osborne 2018; Wakkee et al. 2019), and sharing knowledge (Aluko and Okuwa 2018; Chang 2022; Neary and Osborne 2018) to volunteering of HEIs’ staff in the community (Martínez-Acosta et al. 2023; Shiel et al. 2016).

#### Challenges and drivers

The main challenge to engage in RST refers to a lack of resources and is understood as a lack of time to engage in time-intense HEI–region collaborations (Agusdinata 2022; Leal Filho et al. 2019; Mancini et al. 2022; Purcell et al. 2019; Zilahy and Huisingh 2009), a lack of financial resources including a lack of suitable continuous funding programs (Agusdinata 2022; Aluko and Okuwa 2018; Bodorkós and Pataki 2009; Rosenberg Daneri et al. 2015; Trencher et al. 2014a) and a lack of personnel which refers in particular to the fluctuation of HEIs’ personnel (Agusdinata 2022; Shiel et al. 2016). In addition to this, many activities in the field of sustainability are not institutionalized nor funded and rely on enthusiastic academics (Málovics et al. 2022) who are often challenged to maintain regional partners’ commitment (Agusdinata 2022) and who are confronted with human resource constraints (Quest et al. 2019).

Besides the lack of resources, several studies point to the tensions between academic career demands and engagement in RST as a main challenge—or even barrier—to HEI–region collaboration (Trencher et al. 2014a; Velasco et al. 2021). HEI–region collaboration is not only seen in contradiction to academic performance criteria but to also the self-understanding of HEIs (Mancini et al. 2022), often provoking a discipline-centric mindset (Rosenberg Daneri et al. 2015). The lack of evaluation of HEI–region collaborations in the context of RST further inhibits the interest to engage in this form of collaboration as the effects of these forms of interventions/collaborations are not visibly (Quest et al. 2019; Shiel et al. 2016). Often also a lack of contact and access to suitable regional partners (Leal Filho et al. 2019) inhibits joint efforts toward RST. Within collaboration in RST, it is furthermore emphasized that the involved academic and societal actors are challenged by finding a *balance between societal demands and scientific needs*, including the handling of unequal power relations (Hoinle et al. 2021; Pflitsch and Radinger-Peer 2018; Zilahy and Huisingh 2009) as well as overcoming communication barriers (Agusdinata 2022; Mancini et al. 2022; Zilahy and Huisingh 2009).

As the most prevalent *driver* of HEI–region collaborations in RST, *change agents* are mentioned, referring to engaged academics in key positions (Anderson et al. 2013; Chang 2022; Croese and Duminy 2023; Leal Filho et al. 2019; Pflitsch and Radinger-Peer 2018; Purcell et al. 2019), engaged regional actors (Bodorkós and Pataki 2009; Málovics et al. 2022), students (Agusdinata 2022; Sedlacek 2013; Shiel et al. 2016), ‘bridging organizations’ (Sedlacek 2013; Trencher et al. 2013) as well as a combination of them (Trencher et al. 2013)​. Mancini et al. (2022), Sedlacek (2013) and Wakkee et al. (2019), among others, emphasize the role of HEI leadership as an important driver: mid-term and long-term commitment by the HEI leadership is needed to establish institutional set-ups and to allow for HEI top–down support of HEI bottom–up engagement in RST. Doing so helps to overcome the fragmented character of many RST related activities (Pflitsch and Radinger-Peer 2018).

A further driver states attitudes toward collaboration, i.e., the willingness to cooperate with local actors (Hoinle et al. 2021), openness toward new perspectives (Bogedain and Hamm 2020), regional partners’ commitment (Leal Filho et al. 2019), as well as strategic intentions (Trencher et al. 2013). In this vein, further enabling conditions identified in the literature are perceived mutual benefits (Hoinle et al. 2021; Mancini et al. 2022), raised (business) demands (Purcell et al. 2019), and identified common interest (Bogedain and Hamm 2020). Stated as a challenge before, if not present, a culture of collaboration and emerging networks and trustful relationships can be important drivers of HEI–region collaborations in RST (Hoinle et al. 2021; Leal Filho et al. 2019).

Also, HEI policy from policy makers on national and international levels as well as the availability of pertinent funding programs are significant drivers of HEIs’ engagement in RST (Aluko and Okuwa 2018; Purcell et al. 2019; Trencher et al. 2013, 2014a). On the regime as well as organizational level, it is the institutionalization of new modes of collaboration and interaction (in teaching and research settings) (Bodorkós and Pataki 2009; Málovics et al. 2022)​ as well as changes of curricula and incorporation of sustainability and regional topics into selected courses (Lukman et al. 2009) which spur HEIs’ engagement with RST​.

#### Impact of HEIs in RST

Investigating the role of HEIs in RST, it is of interest how HEIs impact on sustainability in their respective regions. Adopting a broad perspective of sustainability following the internationally agreed upon Sustainable Development Goals (SDGs), HEIs’ impact on regional sustainability is clustered according to the dimensions of sustainability: ecological, social, economic, and governance. As the results of social and economic impacts are closely interlinked, the authors decided to present them together. Moreover, differentiating between impacts and activities is a difficult task, not always feasible based on the data in the sample. The authors therefore present these findings as impacts which were presented as results of activities in the sample.

On the *ecological level*, impacts refer to reductions of emissions or reduction of water and energy consumptions. On the *socio-economic level*, the impact of HEIs on RST is mainly concerning the improvement of livelihood conditions of the local citizens through establishment of income opportunities, providing medical or educational services. On the *governance level* of HEIs’ engagement with RST, HEI start, or support, e.g., the development of local climate or environmental protection plans. A more detailed overview of HEIs’ impacts on RST can be found in Table 2.

**Table 2:** Overview of sustainability impacts of HEIs’ engagement with RST according to SDG sustainability dimensions (own illustration)

Environmental	Socio-economic	Governance
Reductions of carbon emissions (Agusdinata 2022), water consumption (Wakkee et al. 2019) and energy consumption (Leal Filho et al. 2019)	Support (Agusdinata 2022; Shiel et al. 2016) and improvement (Aluko and Okuwa 2018; Bogedain and Hamm 2020; Cobo-Gómez 2024; Leal Filho et al. 2019; Mbah 2019; Perales Franco and McCowan 2021) of local livelihoods especially of women (Aluko and Okuwa 2018; Shiel et al. 2016), local artisans and manufacturers (Cobo-Gómez 2024) and farmers (Mbah 2019)	Development of local climate change policy, climate action plans, building sector policy, energy visions or environmental protection plans (Budowle et al. 2021; Leal Filho et al. 2019; Lukman et al. 2009; Monk et al. 2020; Peer and Stoeglehner 2013; Rosenberg Daneri et al. 2015; Trencher et al. 2014a, b)
Development of technology for cleaning air (Leal Filho et al. 2019)	Establishment of (health) care for the communities (Chang 2022; Perales Franco and McCowan 2021; Purcell et al. 2019)	The alignment of a city’s strategic papers with the SDGs (Croese and Duminy 2023)
Production of organic waste (Leal Filho et al. 2019)	Improving food sovereignty (Mbah 2019; Perales Franco and McCowan 2021)	
Installation of rain gardens on campus to mitigate storm water runoff and protect a local river which is high in biodiversity (Leal Filho et al. 2019)	Providing (life-long) learning opportunities for the region (Monk et al. 2020; Peer and Stoeglehner 2013; Shabalala and Ngcwangu 2021), women (Perales Franco and McCowan 2021), youth (Adamakou et al. 2021; Mdleleni 2022), marginalized communities (Málovics et al. 2022; Neary and Osborne 2018; Nkomo and Sehoole 2007; Perales Franco and McCowan 2021) to increase their capacities	
	Strengthening the political voices of marginalized communities (Málovics et al. 2022; Perales Franco and McCowan 2021)	
	Creation and strengthening civil society networks (Hoinle et al. 2021; Mdleleni 2022)	

## Discussion and further avenues of research

The aim of this paper is to provide insights into the role(s) of HEIs in RST. Conceptualizations and models of HEIs attributing roles to them in societal, sustainable, and regional developments have already been proposed and discussed in the literature (Trippl et al. 2015; Uyarra 2010). However, it has been proposed that the role of HEIs in RST is moving beyond these established role conceptualizations and models [e.g., Trencher et al. (2013, 2014a, b)]. Through a systematic literature review and employing a role theoretical lens based on the operationalization proposed by Wittmayer et al. (2017), the authors analyzed which role(s) of HEIs in RST has/have been theoretically and empirically manifested in the existing literature. To gain deeper insights, the authors specifically looked at the theoretical approaches and conceptualizations used as well as the involved actors, activities, challenges, drivers, and impacts mentioned in the analyzed literature.

As HEIs are large, multi-dimensional and complex with decentralized structures and resulting high levels of autonomy of its individual members (Nieth and Radinger-Peer 2023), one has to take a closer look at whether the role attributed to HEIs in RST is presented as the role of the whole HEI or the role of a specific HEI sub-group (e.g., research group, student group, or HEI leadership). However, this aspect is not made transparent in the analyzed literature which mostly operates on the project level and is then upscaled to reflect on the whole HEI.

An important characteristic in role theory is that roles change over time due to changes in their environment (Turner 1990; Wittmayer et al. 2017). As stated before, the analyzed literature mostly looks at singular projects and not the evolution of HEIs over longer periods of time (exception: Pflitsch and Radinger-Peer 2018). Therefore, it was not possible to derive how the specific role(s) of HEIs in RST has/have changed over time and to answer this research question.

As stated in role theory, roles are inherently context-specific (Biddle 1986; Mead 1934) which in this case is RST. However, as became apparent from the variety of factors influencing the role of HEIs in RST, the deduction of only one role is challenging. The authors therefore refrained from proposing a rigid categorization of distinct roles and rather focused on identifying the essential role of HEIs in RST and different dimensions, through which this role manifests itself.

This literature review shows that this essential role of HEIs in RST is particularly characterized by a holistic consideration of all dimensions of sustainability, rather than merely addressing social, economic, governance or ecological regional needs in a fragmented manner. A main characteristic of the role of HEIs in RST is furthermore the actual implementation of jointly created knowledge. Our review revealed that these processes require collaboration between HEIs and a highly heterogeneous group of regional actors. Particularly noteworthy here is the active engagement of students with regional sustainability challenges and co-creating solutions that became apparent in the sample. However, it needs to be emphasized that although this role of HEIs in RST is beginning to take shape, it also becomes evident that it is not yet institutionally or organizationally embedded within HEIs and largely depends on the commitment of individual actors.

**Fig. 4:**
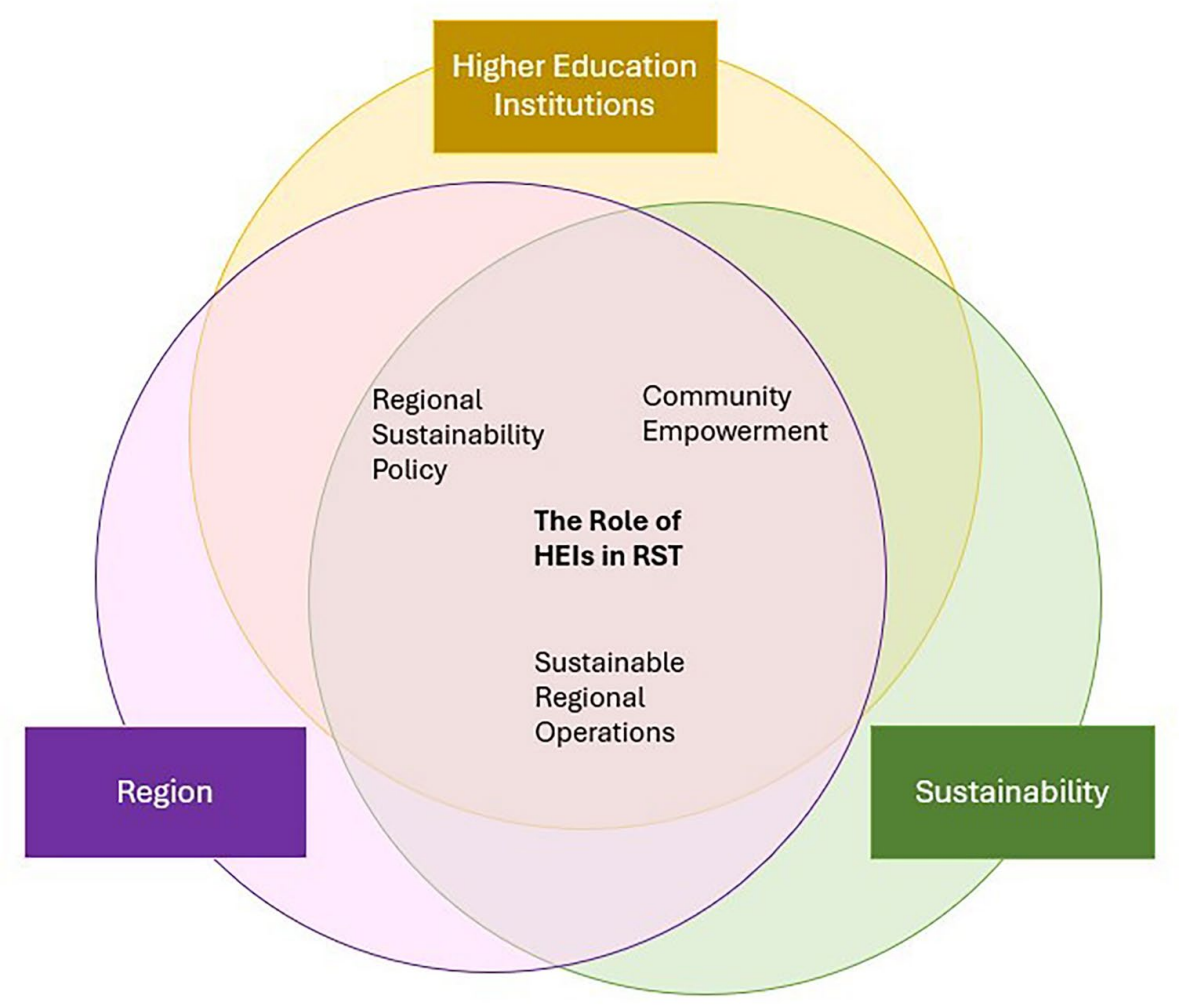
The role of HEIs in RST in its dimensions (own illustration)

Based on the comprehensive results of the systematic literature presented in the section “The role of HEIs in RST: results of the qualitative analysis”, the authors derived three dimensions through which this essential role of HEIs in RST manifests itself, namely Sustainable Regional Operations, Community Empowerment, and Regional Sustainability Policy (see Fig. 4). These dimensions were derived based on the contribution of HEIs to RST (code “impact”). In addition to being the most diverging aspect within the coded material, the authors also follow political (Trippl et al. 2023) and scientific (see, e.g., Trencher et al. 2013, 2014a, b) demands when emphasizing HEIs’ contribution to RST.

### Sustainable regional operations

The dimension *Sustainable Regional Operations* refers to HEIs helping businesses, municipalities, and households to adopt more sustainable operational practices. This includes reducing water and carbon emissions, improving energy and material efficiency, developing emission inventories, creating air-cleaning technologies, promoting cleaner production, and installing community water treatment systems. These are the outcomes of student projects and internships, joint research projects between faculty and regional actors (i.e., public, economic, and civil society), and consulting services from both HEI faculty and students/student organizations in the region. Factors hindering the adoption of this role by HEIs are lack of resources, interest, and skills to engage and the historically difficult relationship between HEIs and regional actors (“ivory tower syndrome”) and, therefore, lacking access to suitable regional partners for cooperation projects. HEIs and especially their faculty also face the challenge of balancing societal demands and scientific needs. However, HEI leadership can play a supportive role through providing resources and putting it on HEIs’ members’ agenda when adapting HEI policies to align with regional needs.

### Community empowerment

The *Community Empowerment* dimension is characterized by its focus on helping to improve livelihood conditions of HEIs’ surrounding communities through establishing new markets, improving production processes, providing (live-long) learning opportunities, strengthening the political voice and increasing the capacities of marginalized communities, strengthening civil society networks, and providing health care services to the community. Again, HEI students and student organizations are engaged through student projects or internships in the community. Moreover, faculty help establish this dimension through hosting bi-/multilateral knowledge exchange workshops, providing policy briefs to (local) authorities and public institutions and adopting certain research practices such as transdisciplinary research or participatory action research where local citizens or civil society organizations are regarded as partners in the creation of solutions for challenges they face. Cooperative research projects are also supported by HEIs’ service centers and students who help set up workshops or gather data. Again, the establishment and adoption of this dimension is dependent on the availability of resources (financial and time) and the personal engagement of HEI and regional actors as the benefit from these cooperations is often not evident, esp. for regional actors. Here, the HEI members are also confronted with the ivory tower syndrome and are often regarded solely as knowledge providers. The lack of trust—also influenced due to high fluctuation of HEI personnel—of regional actors in HEI actors is a hampering factor. HEI policies play an influencing factor as the institutionalization of new modes of collaboration and cooperation in both education and research would spur the establishment of this dimension. While regional actors are hampered by the bureaucratical burden in cooperation projects, HEI actors have to balance scientific and societal demands.

### Regional sustainability policy

The *Regional sustainability policy* dimension refers to HEIs spurring the adoption and implementation of regional government strategies and policies for sustainability^2^ or the (re-)design of policy instruments to advance regional sustainability (e.g., ban on commercial tree cutting or implementation of energy efficiency standards for the building sectors). Moreover, through HEIs’ engagement with regional actors, cooperation agreements between different regional actors are implemented. Again, students are actively engaged with regional actors (i.e., public and civil society) through internships and student projects. Also, collaborative teaching and research projects with regional economic, public, and civil society actors influence regional sustainability policy development. Moreover, HEI faculty and students contribute to regional policies through participating in regional networks, hosting networking events, and providing consulting services. Lack of resources (time and financial), lack of interest and skills to collaborate, fluctuation of HEI personnel, and access to regional partners are inhibiting the adoption of this dimension by HEIs. Moreover, HEIs’ contribution to shaping regional policy toward sustainability is also hampered by regional policy actors who are not open for cooperation. The adoption of this dimension is driven by engaged HEIs actors who are supported by HEI leadership and who can align the HEI policies with regional needs. Moreover, institutionalized cooperation and exchange structures between HEI and regional actors spur this dimension.

As roles change over time due to changes in their environment (Callero 1994; Lynch 2007; Turner 1990) and HEIs are heterogeneous actors, they can enact the dimensions of this essential role simultaneously. This is also visible in the analyzed sample where two (Agusdinata 2022; Chang 2022; Cobo-Gómez 2024; Hoinle et al. 2021; Lukman et al. 2009; Martínez-Acosta et al. 2023; Monk et al. 2020; Peer and Stoeglehner 2013; Pflitsch and Radinger-Peer 2018; Purcell et al. 2019; Rosenberg Daneri et al. 2015; Velasco et al. 2021) or even all three dimensions (Leal Filho et al. 2019) are present at the same time. Moreover, they can also only exhibit one dimension of this role at a certain point in time. The adoption of these dimensions and generally a role in RST is influenced by HEI-internal (e.g., agenda setting of HEI leadership, internal funding opportunities) and external [e.g., societal and political demands for HEIs to contribute to sustainability (Kohl et al. 2022), integration of sustainability-related criteria into performance agreements, awards, and ranking schemes (Atici et al. 2021)] factors and developments.

Further avenues of research are the investigation of who (from both the regional and HEI side) decides whether an HEI takes on the role of contributing to RST, which dimension(s) is/are enacted and whether the desired role or dimension can be imposed top-down due to the high autonomy of HEI members. Moreover, HEI–region settings are place-specific and thus unique, making the investigation of, e.g., historical paths, actor constellations, incentives, and barriers essential for understanding the role of HEI in a particular RST and why this role might differ from other HEI–region settings which seem, at a glance, to be similar. The sample literature mainly looks at the role of HEIs in RST at a certain point in time and disregards the evolution over time (exception: Pflitsch and Radinger-Peer 2018). However, role theory highlights the dynamic characteristics of roles, as they change when social structures and the environment around them change (Callero 1994; Turner 1990). Therefore, one avenue of further research is the investigation of the evolution of HEIs engagement in RST over time to decipher when, why, and how they engage in more detail. A closer investigation of the HEIs and regional actors who are driving the engagement of HEIs with RST and their motivation, visions, resources, and positions is also still pending.

## Conclusion

The role of HEIs in society as institutions has undergone numerous transformations since their establishment in the eleventh century (Scholz 2020). As awareness of global problems has increased, so too has the demand on HEIs to contribute to understanding and solving them. Moreover, in recent years, the interconnectedness of the global problems with their local manifestations has been widely discussed, highlighting the importance of local solutions which are co-created by local actors (Dlouhá et al. 2013). As HEIs are important actors in RST, the main theoretical contribution of this paper is the systematic conceptualization of the role of HEIs in RST and here more specifically the application of role theory to HEIs which are characterized by both individual and collective agency. Building on this theoretical implication, the paper offers a set of practical implications which relate to the importance of different types of individual and institutional actors and answers practical questions like which actors are involved in RST, who initiates and how are the actors interrelated. Here the paper identifies three dimensions (sustainable regional operations, community empowerment, and regional sustainability policy) along which HEIs are interrelated with other regional actors, and which characterize HEIs’ specific role in RST. Knowledge about the characteristics of these three dimensions can be used pro-actively by both HEIs and regions in organizing and coordinating activities for RST.

## Data Availability

The raw material of the search process, final selection for inclusion in the literature review and the coded material used in this study is available in Zenodo repository [10.5281/zenodo.17036808].

## Appendices

### Appendix A—overview of the analyzed literature

Adamakou, M., Niavis, S., Kallioras, D., & Petrakos, G. (2021). Understanding the Regional Engagement of Universities from a Stakeholders’ Perspective: The Case of the University of Thessaly, Greece. Sustainability, 13(19), 10565. 10.3390/su131910565.

Agusdinata, D. B. (2022). The role of universities in SDGs solution co-creation and implementation: A human-centered design and shared-action learning process. Sustainability Science, 17(4), 1589–1604. 10.1007/s11625-022-01128-9.

Aluko, Y. A., & Okuwa, O. B. (2018). Innovative solutions and women empowerment: Implications for sustainable development goals in Nigeria. African Journal of Science, Technology, Innovation and Development, 10(4), 441–449. 10.1080/20421338.2018.1471028.

Anderson, P. M. L., Brown-Luthango, M., Cartwright, A., Farouk, I., & Smit, W. (2013). Brokering communities of knowledge and practice: Reflections on the African Centre for Cities’ CityLab programme. Cities, 32, 1–10. 10.1016/j.cities.2013.02.002.

Bodorkós, B., & Pataki, G. (2009). Linking academic and local knowledge: Community-based research and service learning for sustainable rural development in Hungary. Journal of Cleaner Production, 17(12), 1123–1131. 10.1016/j.jclepro.2009.02.023.

Bogedain, A., & Hamm, R. (2020). Strengthening local economy – an example of higher education institutions’ engagement in “co-creation for sustainability.” REGION, 7(2), 9–27. 10.18335/region.v7i2.271.

Budowle, R., Krszjzaniek, E., & Taylor, C. (2021). Students as Change Agents for Community–University Sustainability Transition Partnerships. Sustainability, 13(11), 6036. 10.3390/su13116036.

Chang, H. B. (2022). Developing Companionship with the Left-Behinds: University Social Responsibility and a Collaborative Approach to Rural Regeneration in the Badlands Region of Taiwan. Transactions of the Association of European Schools of Planning, 6(1), 14–29. 10.24306/TrAESOP.2022.01.002.

Cobo-Gómez, J. C. (2024). Social innovation in university-community partnerships in Latin America: Exploring collaborative models. Sustainable Technology and Entrepreneurship, 3(2), 100,061. 10.1016/j.stae.2023.100061.

Croese, S., & Duminy, J. (2023). Co-producing urban expertise for SDG localization: The history and practices of urban knowledge production in South Africa. Urban Geography, 44(3), 538–557. 10.1080/02723638.2022.2079868.

Hoinle, B., Roose, I., & Shekhar, H. (2021). Creating Transdisciplinary Teaching Spaces. Cooperation of Universities and Non-University Partners to Design Higher Education for Regional Sustainable Transition. Sustainability, 13(7), 3680. 10.3390/su13073680.

Leal Filho, W., Caughman, L., Pimenta Dinis, M. A., Frankenberger, F., Azul, A. M., & Salvia, A. L. (2022). Towards symbiotic approaches between universities, sustainable development, and cities. Scientific Reports, 12(1), 11,433. 10.1038/s41598-022-15717-2.

Leal Filho, W., Vargas, V. R., Salvia, A. L., Brandli, L. L., Pallant, E., Klavins, M., Ray, S., Moggi, S., Maruna, M., Conticelli, E., Ayanore, M. A., Radovic, V., Gupta, B., Sen, S., Paço, A., Michalopoulou, E., Saikim, F. H., Koh, H. L., Frankenberger, F., … Vaccari, M. (2019). The role of higher education institutions in sustainability initiatives at the local level. Journal of Cleaner Production, 233, 1004–1015. 10.1016/j.jclepro.2019.06.059.

Lehmann, M., Christensen, P., Thrane, M., & Jørgensen, T. H. (2009). University engagement and regional sustainability initiatives: Some Danish experiences. Journal of Cleaner Production, 17(12), 1067–1074. 10.1016/j.jclepro.2009.03.013.

Lukman, R., Krajnc, D., & Glavič, P. (2009). Fostering collaboration between universities regarding regional sustainability initiatives – the University of Maribor. Journal of Cleaner Production, 17(12), 1143–1153. 10.1016/j.jclepro.2009.02.018.

Mader, C., Zimmermann, F. M., Steiner, G., & Risopoulos, F. (2008). Regional Centre of Expertise (RCE) Graz‐Styria: A process of mobilization facing regional challenges. International Journal of Sustainability in Higher Education, 9(4), 402–415. 10.1108/14676370810905517.

Málovics, G., Juhász, J., & Bajmócy, Z. (2022). The potential role of university-community engagement (UCE) in social justice and sustainability transformation—The case of the University of Szeged (Hungary). DETUROPE—The Central European Journal of Tourism and Regional Development, 14(3), 103–128. 10.32725/det.2022.024.

Mancini, M. C., Arfini, F., & Guareschi, M. (2022). When Higher Education Meets Sustainable Development of Rural Areas: Lessons Learned from a Community–University Partnership. Social Sciences, 11(8), 338. 10.3390/socsci11080338.

Marchigiani, E., & Garofolo, I. (2023). Italian Universities for Territorial Sustainable Development and Responsible Communities—The Case Study of the University of Trieste. Sustainability, 15(3), 2325. 10.3390/su15032325.

Martínez-Acosta, M., Vázquez-Villegas, P., Mejía-Manzano, L. A., Soto-Inzunza, G. V., Ruiz-Aguilar, K. M., Kuhn Cuellar, L., Caratozzolo, P., & Membrillo-Hernández, J. (2023). The implementation of SDG12 in and from higher education institutions: Universities as laboratories for generating sustainable cities. Frontiers in Sustainable Cities, 5, 1,158,464. 10.3389/frsc.2023.1158464.

Mbah, M. (2019). Can local knowledge make the difference? Rethinking universities’ community engagement and prospect for sustainable community development. The Journal of Environmental Education, 50(1), 11–22. 10.1080/00958964.2018.1462136.

Mdleleni, L. (2022). University as a vehicle to achieve social innovation and development: Repositioning the role of the university in the society. Social Enterprise Journal, 18(1), 121–139. 10.1108/SEJ-10-2020-0093.

Monk, D., Openjuru, G., Odoch, M., Nono, D., & Ongom, S. (2020). When the guns stopped roaring: Acholi ngec ma gwoko lobo. Gateways: International Journal of Community Research and Engagement, 13(1). 10.5130/ijcre.v13i1.7194.

Neary, J., & Osborne, M. (2018). University engagement in achieving sustainable development goals: A synthesis of case studies from the SUEUAA study. Australian Journal of Adult Learning, 58(3), 336–364.

Nkomo, M., & Sehoole, C. (2007). Rural-based universities in South Africa Albatrosses or potential nodes for sustainable development? Int J Sustain High Educ. 10.1108/14676370710726689

Paunović, I., Müller, C., & Deimel, K. (2022). Building a Culture of Entrepreneurial Initiative in Rural Regions Based on Sustainable Development Goals: A Case Study of University of Applied Sciences–Municipality Innovation Partnership. Sustainability, 14(19), 12,108. 10.3390/su141912108.

Peer, V., & Stoeglehner, G. (2013). Universities as change agents for sustainability – framing the role of knowledge transfer and generation in regional development processes. Journal of Cleaner Production, 44, 85–95. 10.1016/j.jclepro.2012.12.003.

Perales Franco, C., & McCowan, T. (2021). Rewiring higher education for the Sustainable Development Goals: The case of the Intercultural University of Veracruz, Mexico. Higher Education, 81(1), 69–88. 10.1007/s10734-020-00525-2.

Pflitsch, G., & Radinger-Peer, V. (2018). Developing Boundary-Spanning Capacity for Regional Sustainability Transitions—A Comparative Case Study of the Universities of Augsburg (Germany) and Linz (Austria). Sustainability, 10(4), 918. 10.3390/su10040918.

Purcell, W. M., Henriksen, H., & Spengler, J. D. (2019). Universities as the engine of transformational sustainability toward delivering the sustainable development goals: “Living labs” for sustainability. International Journal of Sustainability in Higher Education, 20(8), 1343–1357. 10.1108/IJSHE-02-2019-0103.

Quest, J., Shiel, C., & Watson, S. (2019). Transitioning toward a sustainable food city. International Journal of Sustainability in Higher Education, 20(7), 1258–1277. 10.1108/IJSHE-09-2018-0159

Ramos, T. B. (2009). Development of regional sustainability indicators and the role of academia in this process: The Portuguese practice. Journal of Cleaner Production, 17(12), 1101–1115. 10.1016/j.jclepro.2009.02.024.

Rosenberg Daneri, D., Trencher, G., & Petersen, J. (2015). Students as change agents in a town-wide sustainability transformation: The Oberlin Project at Oberlin College. Current Opinion in Environmental Sustainability, 16, 14–21. 10.1016/j.cosust.2015.07.005.

Sedlacek, S. (2013). The role of universities in fostering sustainable development at the regional level. Journal of Cleaner Production, 48, 74–84. 10.1016/j.jclepro.2013.01.029.

Shabalala, L. P., & Ngcwangu, S. (2021). Accelerating the implementation of SDG 4: Stakeholder perceptions toward initiation of sustainable community engagement projects by higher education institutions. International Journal of Sustainability in Higher Education, 22(7), 1573–1591. 10.1108/IJSHE-04-2020-0123.

Shiel, C., Leal Filho, W., Paço, A., & Brandli, L. (2016). Evaluating Universities Engagement in Capacity Building for Sustainable Development in Local Communities. Evaluation and Program Planning, 54, 123–134. 10.1016/j.evalprogplan.2015.07.006

Trencher, G., Bai, X., Evans, J., McCormick, K., & Yarime, M. (2014a). University partnerships for co-designing and co-producing urban sustainability. Global Environmental Change, 28, 153–165. 10.1016/j.gloenvcha.2014.06.009.

Trencher, G., Yarime, M., & Kharrazi, A. (2013). Co-creating sustainability: Cross-sector university collaborations for driving sustainable urban transformations. Journal of Cleaner Production, 50, 40–55. 10.1016/j.jclepro.2012.11.047.

Trencher, G., Yarime, M., McCormick, K. B., Doll, C. N. H., & Kraines, S. B. (2014b). Beyond the third mission: Exploring the emerging university function of co-creation for sustainability. Science and Public Policy, 41(2), 151–179. 10.1093/scipol/sct044.

Velasco, D., Boni, A., Delgado, C., & Rojas-Forero, G. D. (2021). Exploring the Role of a Colombian University to Promote Just Transitions. An Analysis from the Human Development and the Regional Transition Pathways to Sustainability. Sustainability, 13(11), 6014. 10.3390/su13116014.

Velázquez, L., Munguía, N., Zavala, A., & Navarrete, M. D. L. Á. (2008). Challenges in operating sustainability initiatives in Northwest Mexico. Sustainable Development, 16(6), 401–409. 10.1002/sd.357.

Wakkee, I., Van Der Sijde, P., Vaupell, C., & Ghuman, K. (2019). The university’s role in sustainable development: Activating entrepreneurial scholars as agents of change. Technological Forecasting and Social Change, 141, 195–205. 10.1016/j.techfore.2018.10.013.

Zilahy, G., & Huisingh, D. (2009). The roles of academia in Regional Sustainability Initiatives. Journal of Cleaner Production, 17(12), 1057–1066. 10.1016/j.jclepro.2009.03.018.

### Appendix B

Table 3 gives an overview on all outlets which only published one text which was included in this review.

**Table 3:** Overview of all journals where only one published paper was included in this review (own illustration)

Scientific Reports	African Journal of Science, Technology, Innovation and Development
Australian Journal of Adult Learning	Evaluation and Program Planning
Current Opinion in Environmental Sustainability	Cities
Higher Education	Technological Forecasting & Social Change
Region	Review Regional Research
Science and Public Policy	Social Enterprise Journal
Social Sciences	Sustainability Science
Sustainable Development	Sustainable Technology and Entrepreneurship
International Journal of Community Research and Engagement	The Central European Journal of Regional Development and Tourism
The Journal of Environmental Education	Urban Geography
Transactions of the Association of European Schools of Planning	Frontiers in Sustainable Cities; Global Environmental Change

Table 4 depicts all involved actors with the associated literature.

**Table 4:** Overview of HEI and regional actors mentioned in the literature sample (own illustration)

HEIs	Region
*Students* (Adamakou et al. 2021; Agusdinata 2022; Bodorkós and Pataki 2009; Bogedain and Hamm 2020; Budowle et al. 2021; Chang 2022; Cobo-Gómez 2024; Hoinle et al. 2021; Leal Filho et al. 2019, 2022; Lehmann et al. 2009; Lukman et al. 2009; Mader et al. 2008; Málovics et al. 2022; Mancini et al. 2022; Marchigiani and Garofolo 2023; Martínez-Acosta et al. 2023; Mbah 2019; Monk et al. 2020; Paunović et al. 2022; Perales Franco and McCowan 2021; Quest et al. 2019; Ramos 2009; Rosenberg Daneri et al. 2015; Sedlacek 2013; Shiel et al. 2016; Trencher et al. 2014a; Velasco et al. 2021; Velázquez et al. 2008; Zilahy and Huisingh 2009) and *student organizations* (Budowle et al. 2021; Chang 2022; Lukman et al. 2009; Mader et al. 2008; Wakkee et al. 2019)	*Public* (Agusdinata 2022; Anderson et al. 2013; Bodorkós and Pataki 2009; Budowle et al. 2021; Chang 2022; Cobo-Gómez 2024; Croese and Duminy 2023; Leal Filho et al. 2022; Lehmann et al. 2009; Lukman et al. 2009; Mader et al. 2008; Marchigiani and Garofolo 2023; Martínez-Acosta et al. 2023; Monk et al. 2020; Paunović et al. 2022; Peer and Stoeglehner 2013; Pflitsch and Radinger-Peer 2018; Quest et al. 2019; Ramos 2009; Rosenberg Daneri et al. 2015; Shiel et al. 2016; Trencher et al. 2014a, b; Velasco et al. 2021; Velázquez et al. 2008), e.g., administration, government, institutions (e.g., schools, church, libraries, hospital)
*Faculty* (Agusdinata 2022; Chang 2022; Cobo-Gómez 2024; Leal Filho et al. 2019, 2022; Lehmann et al. 2009; Lukman et al. 2009; Marchigiani and Garofolo 2023; Martínez-Acosta et al. 2023; Mbah 2019; Peer and Stoeglehner 2013; Perales Franco and McCowan 2021; Trencher et al. 2014a, b; Trencher et al. 2013; Velasco et al. 2021; Wakkee et al. 2019; Zilahy and Huisingh 2009), *research groups* (Anderson et al. 2013; Bodorkós and Pataki 2009; Bogedain and Hamm 2020; Croese and Duminy 2023; Mader et al. 2008; Málovics et al. 2022; Mancini et al. 2022; Monk et al. 2020; Neary and Osborne 2018; Paunović et al. 2022; Pflitsch and Radinger-Peer 2018; Velázquez et al. 2008) and HEIs teachers (Hoinle et al. 2021; Leal Filho et al. 2019, 2022; Mbah 2019)	*Private* (Agusdinata 2022; Anderson et al. 2013; Cobo-Gómez 2024; Leal Filho et al. 2022; Lehmann et al. 2009; Lukman et al. 2009; Mancini et al. 2022; Mbah 2019; Paunović et al. 2022; Purcell et al. 2019; Quest et al. 2019; Rosenberg Daneri et al. 2015; Shiel et al. 2016; Trencher et al. 2014b; Velázquez et al. 2008), e.g., companies, industry
*HEI management* (Paunović et al. 2022; Velasco et al. 2021) and *leadership* (Agusdinata 2022; Cobo-Gómez 2024; Trencher et al. 2014b; Velasco et al. 2021), *HEI administration* (Lehmann et al. 2009; Mader et al. 2008; Paunović et al. 2022; Sedlacek 2013; Trencher et al. 2014a; Velasco et al. 2021) and *HEI service centers* (Budowle et al. 2021; Lehmann et al. 2009; Lukman et al. 2009; Mader et al. 2008; Mancini et al. 2022; Paunović et al. 2022; Purcell et al. 2019; Wakkee et al. 2019), e.g., knowledge transfer office, sustainability committees/councils	*Civil society* (Agusdinata 2022; Aluko and Okuwa 2018; Anderson et al. 2013; Bodorkós and Pataki 2009; Cobo-Gómez 2024; Leal Filho et al. 2019, 2022; Lukman et al. 2009; Málovics et al. 2022; Martínez-Acosta et al. 2023; Mbah 2019; Mdleleni 2022; Monk et al. 2020; Neary and Osborne 2018; Peer and Stoeglehner 2013; Perales Franco and McCowan 2021; Quest et al. 2019; Rosenberg Daneri et al. 2015; Sedlacek 2013; Shiel et al. 2016; Velázquez et al. 2008), e.g., citizens, local community, NGOs, networks, associations
*Alumni* (Velasco et al. 2021)	*Foundations* (Aluko and Okuwa 2018; Bodorkós and Pataki 2009; Mancini et al. 2022; Velasco et al. 2021)
*RCEs* (if positioned at an HEI) (Sedlacek 2013)	

## Footnotes

1 The term “institution” in HEI has a double meaning: Due to their long history and their societal status, HEIs are regarded as societal institutions characterized by the presence of established rules, organized practices, values, and norms that inform their activities. Moreover, they are understood as societal organizations which considers how they are organized to achieve its tasks and objectives (Olsen 2007). In the following, the authors use the term “institution” to refer to the organization in its institutional context.

2 The term sustainability refers to its four dimensions, i.e., environmental, economic, social, and governance.
